# Controlling Tsetse Flies and Ticks Using Insecticide Treatment of Cattle in Tororo District Uganda: Cost Benefit Analysis

**DOI:** 10.3389/fvets.2021.616865

**Published:** 2021-03-22

**Authors:** Walter O. Okello, Ewan T. MacLeod, Dennis Muhanguzi, Charles Waiswa, Susan C. Welburn

**Affiliations:** ^1^Infection Medicine, Deanery of Biomedical Sciences, College of Medicine and Veterinary Medicine, The University of Edinburgh, Edinburgh, United Kingdom; ^2^Commonwealth Scientific and Industrial Research Organisation, Black Mountain Science and Innovation Park, Canberra, ACT, Australia; ^3^Department of Biomolecular and Biolaboratory Sciences, School of Biosecurity, Biotechnical and Laboratory Sciences, College of Veterinary Medicine Animal Resources and Biosecurity, Makerere University, Kampala, Uganda; ^4^The Coordinating Office for Control of Trypanosomiasis in Uganda, Kampala, Uganda; ^5^Zhejiang University–University of Edinburgh Institute, Zhejiang University, International Campus, Haining, China

**Keywords:** tick control, cost–benefit analysis, Uganda, trypanosomiasis, gross margin analysis

## Abstract

**Background:** The endemic vector-borne diseases transmitted by tsetse and ticks impose heavy burdens on the livestock keepers in Africa. Applying deltamethrin to the belly, legs, and ears of cattle offers a possibility of mitigating these losses at a cost affordable to livestock keepers. Although studies have quantified the impacts of individual diseases on livestock productivity, little is known about the dual economic benefits of controlling both tsetse and ticks, nor about the number of cattle that need to be treated to confer these benefits. Alongside an epidemiological study in south-east Uganda, a farm level assessment was done to investigate the benefits and costs of spraying different proportions of the village cattle population using this restricted application protocol.

**Methods:** A study comprising 1,902 semi-structured interviews was undertaken over a period of 18 months. Financial data on household income and expenditure on cattle was collected, and cost-benefit analysis was done pre- and post-intervention and for different spraying regimes. The total cost of the intervention was obtained from the implementation costs of the epidemiological study and from expenses incurred by participating farmers enabling examination of benefit-cost ratios and incremental benefit-cost ratios for each treatment regime.

**Results:** The benefit-cost analysis of spraying 25%, 50%, and 75% of the cattle population yielded average benefit-cost ratios of 3.85, 4.51, and 4.46. The incremental benefit-cost ratios from spraying each additional 25% of the cattle population were 11.38, 3.89, and 0.79, showing a very high return on investment for spraying 50% of the population, with returns reducing thereafter.

**Conclusion:** Comparing the gross margins per bovine, the study found that increasing the proportion of cattle sprayed yielded increasing benefits to the farmers, but that these benefits were subject to diminishing returns. From a practical viewpoint, this study recommends spraying only draft cattle to control trypanosomiasis and tick-borne diseases in this area as they make 38.62% of the cattle population, approaching the 50% threshold. In areas with a lower proportion of draft males, farmers could be advised to also include cows.

## Background

Animal African trypanosomiasis (AAT) can be controlled by targeting the tsetse fly or by using chemotherapeutic/chemoprophylactic trypanocidal drugs (1, 2). Approaches are often combined in order to improve the effectiveness of control measures (3). To control tsetse flies, ground spraying of tsetse breeding and resting sites with residual insecticides and aerial spraying have both been deployed in Uganda in the past (3, 4). The drawbacks to large scale spraying of tsetse habitats include environmental degradation, relatively high costs, and the unsuitability of aerial spraying for hilly terrain (5). Environmental issues associated with large scale spraying led to the development of bait technologies that can be implemented by the community. Stationary baits include traps and insecticide-impregnated targets, both of which may also be odor-baited, and mobile baits, in the form of insecticide-treated cattle (ITC) on which insecticide can be applied by dipping, spraying, or pouring-on formulations (6). Traps have been successfully deployed in Uganda to control human African trypanosomiasis during epidemics (7).

The restricted application protocol (RAP) is a refinement of ITC that involves spraying of insecticide at dip concentration only to the tsetse predilection feeding sites on cattle (the legs and belly), rather than applying insecticide to the entire animal (as in dipping) or when a concentrated formula is applied along the back of the animal in the case of pour-on formulations (8). Controlling tsetse in this way offers benefits that go beyond reducing the incidence of trypanosomiasis in livestock.

Throughout Eastern Africa, livestock keepers have sought to mitigate the impacts of tick-borne infections, especially *Theileria parva* (East Coast fever). Although an “infect and treat vaccine” for *T. parva* exists, this has not been widely adopted in Uganda. Livestock keepers buy acaricides which are applied to their animals by spraying or pouring-on, more rarely by dipping. These acaricides are not effective against tsetse; however, deltamethrin is effective against ticks as well as tsetse (9). By including the ears of the cattle, a tick-predilection site, to the RAP spraying regime, it is possible to recommend this to farmers to replace their usual acaricide treatments. RAP is environmentally friendly and cheaper as it uses less insecticide compared to other methods, while conserving the enzootic stability of tick-borne diseases (TBDs) (10). Lastly, south-eastern Uganda, including Tororo District, is a focus of the *Trypanosoma brucei rhodesiense* form of human African trypanosomiasis (rHAT) which is a zoonosis. Thus, measures to control the tsetse population will also reduce the transmission of rHAT to humans (11).

Aside from vector control, trypanocidal drugs are deployed prophylactically and therapeutically for AAT control. It has been estimated that 35 million doses of trypanocides were administered annually in Africa (12). Control of TBDs and AAT is often considered to be a private good with the farmers frequently expected to pay for the service (9). To ensure the participation of farmers and decrease overall disease impacts, it is vital to have an affordable and effective farmer-led integrated control of both tsetse and TBDs (13).

Assessments of the costs and benefits of controlling tsetse and trypanosomiasis (14–16) are critical for deciding on interventions. Uganda was the subject of one of the earliest studies on the economics of AAT control (17). More recently the costs of different techniques for tsetse control were estimated for Uganda (18); furthermore, an analysis of historic and current tsetse control costs was undertaken (19). The few recent studies that looked at the impact of trypanosomiasis in Uganda include assessment of the economic impact of bovine trypanosomosis (20) and socio-economic and livestock survey of agro-pastoral communities (21), the latter emphasizing the importance of draft power, which was analyzed in more detail in Tororo District (22).

The largest scale study on the impact of AAT was undertaken by the African Trypanotolerant Livestock Network (15, 23). Similar protocols were implemented in study sites in eight countries, combining the monitoring of cattle, sheep, and goats for trypanosomiasis alongside the collection of livestock productivity data, including milk yields (23). Productivity data from various studies have been used to model the economic impact of AAT and its control in different contexts (24–26). Field studies, except for the African Trypanotolerant Livestock Network studies, have largely been cross- sectional and under-powered with relatively small sample sizes. Obtaining the information required for these analyses is challenging because cattle productivity is difficult to measure, farm level interviews are time-consuming, and the inclusion of longitudinal studies increases project costs.

Studies have almost exclusively directly measured or focused on cattle production parameters (such as deaths, births, weight, and milk yields) rather than on the effects on the incomes of livestock keepers. Defining a comparator has also been challenging. These have often been based on comparing households or animals who participated in trypanosomiasis control activities (usually referred to as “with” intervention) to those who did not (“without” intervention). Other counterfactual estimates have been based on measured prevalence (23) but in an era before the use of molecular techniques or comparison of before and after situations without a control (no intervention).

For ITC, there has been little information about the optimal proportion or number of cattle to be sprayed in order to control trypanosomiasis, and therefore calculations have been based on estimates (18). However, in 2014, Kajunguri et al. calculated that, using RAP where 27% of the cattle herd was treated, was sufficient to control *Trypanosoma. brucei sensu lato* (27). Field studies were undertaken to confirm the modeling work and showed that spraying 25% of cattle using RAP could be effective in preventing re-infection of cattle with trypanosomes in south-east Uganda, protecting humans from rHAT as well as controlling AAT (28). In south-east Uganda, it has been shown that cattle act as the main reservoir for rHAT (13, 29–31). A study in Dokolo and Kaberamaido districts in south-east Uganda found that injection of cattle with the trypanocide diminazene aceturate followed by the use of RAP reduced the prevalence of *Trypanosoma vivax* from 5.9 to 0.5% in cattle (32).

Our study aimed to gain an understanding of the impact of RAP by quantifying its costs and benefits from the societal perspective, examining how these varied across differing proportions of the cattle population, and looking at broader implications of the findings for effective disease control. Specific questions were: (i) what is the net benefit of spraying additional proportion of the cattle population? and (ii) what is the benefit-cost ratio for the different proportions of the cattle population sprayed using RAP?

## Methods

### Selection of Study Villages, Households and Data Collection

The intervention was undertaken from June 2012 to December 2013 in Tororo District, south-east Uganda. Tororo is semi-arid where small-holder crop-livestock systems dominate. There are over 400 villages keeping more than 37,000 cattle, most of which are male used for draft work, usually referred to as work oxen, both castrated or uncastrated (33). The selection of villages for the economic study was based on a concurrent epidemiological study evaluating the effectiveness of controlling trypanosomiasis by spraying different proportions of the cattle population using RAP (28).

Twenty villages met the criteria for inclusion in the epidemiological study (28). The criteria were cattle AAT prevalence of 15% or more, a population of 50 or more cattle in the village, and villages being at least 5 km apart.

The 20 selected villages were randomly allocated to five RAP treatments (28). All cattle were given two standard doses of Veriben B12 [CEVA Santé Animale, containing diminazene aceturate, vitamin B12 (cyanocobalamin) and B12a (hydroxocobalamin)] forty days apart to treat any pre-existing trypanosome infection (28, 34). Ver Veriben Treatment 1 (T1) cattle received no further treatments whereas treatments 2 to 4 (i.e., T2, T3, and T4) consisted of spraying 25, 50, and 75% of the village cattle population using RAP at 28 days intervals for 18 months. Treatment 5 (T5) comprised of villages whose cattle were dewormed once every 3 months but received no other treatment. T1 and T5 were considered as control groups for the graded (25, 50, and 75%) RAP regimes (28). In order not to complicate the results by having to allow for the benefits from deworming, for this economic study, the T1 villages were used as the control for comparing benefit-cost ratios as well as calculating the incremental cost benefit ratios.

The sample size (number of households to be studied) was determined using CSurvey software version 2.0 (35) where the expected prevalence of AAT was set as 15%, rate of homogeneity at 0.15 and the average eligible persons per household as 1. A sample size of 600 households (30 for each village) was calculated for the economic baseline survey. Within the selected villages a full list of cattle-owning households had already been compiled and updated *via* a cattle register, at the start of the epidemiological study (28). A subset of households was selected from the updated cattle register for the economic study using random selection, to avoid selection bias, with the starting household selected using a spin dial (35); a feature in CSurvey software that generates a random direction, hence a random starting household. Use of the same cattle register ensured that the two studies are based on the same households and thus the data were consistent.

Prior to data collection, two study enumerators were given 10 days training on how to approach farmers, to identify study cattle (using ear tag numbers) and to complete questionnaires appropriately. Enumerators were deployed to villages depending on their understanding of the local language with each covering 10 villages. Semi-structured questionnaires were pre-tested in two villages that were not included among those randomly selected for this study. The information recorded included whether the cattle in the household had had a blood sample taken by the epidemiology team, household characteristics (number of people, livestock kept, type of dwelling, and animal health measures used) and components of cattle-related income and expenditure.

Excluding T5, data regarding the cost and benefit of RAP were collected from 480 households in the 16 villages eligible for RAP intervention using 6 months recall over a period of 18 months (a total of 1,920 interviews before and during the RAP intervention) to minimize recall bias (36). Six months recall enabled data on cattle “exits” and “entries,” productivity, mortality, fertility, cattle-related expenses and revenues, the number of times farmers took their cattle for spraying, and the related costs to the household to be updated. The data were entered, cleaned and analdata were entered, cleaned, and anayzed in Microsoft Excel® 2013.

### Economic Analysis

To determine the efficiency of different strategies to control trypanosomiasis and TBDs using insecticide-treated cattle, we used cost-benefit analysis and marginal analysis (37). Unlike cost-effectiveness analysis where monetary and non-monetary aspects of a health intervention can be combined (38), all values in this study were monetary, enabling the use of cost-benefit analysis. Cost-benefit analysis was used to derive benefit-cost ratios, incremental benefit-cost ratios, and net benefit. Benefit-cost ratios were computed using the standard method of dividing benefits by the cost (26) of each treatment regime. Incremental benefit-cost analysis was analyzed using Equation 1, which involved (1) arranging the RAP treatment regimes in ascending order based on cost, (2) subtracting the total benefits of the treatment regimens being compared to obtain marginal benefit, (3) subtracting the total cost of the treatment regimens being compared to obtain marginal cost, and (4) dividing the marginal benefit with the marginal cost.

(1) $$\begin{array}{r} IBCR=\sum B_{1}-\sum B_{2}/\sum C_{1}-\sum C_{2} \end{array}$$

Where, *IBCR* is incremental benefit-cost ratio, ∑B_1_ is the total benefit of RAP for treatment 1 (i.e., base comparator), ∑B_2_ is the benefit of RAP for treatment 2 (i.e., the challenger), ∑*C*_1_ is the total cost of RAP for treatment 1 (i.e., base comparator), and ∑*C*_2_ is the total cost of RAP for treatment 2 (i.e., the challenger). Because the treatments had different total cost, the benefit-cost ratios were adjusted to ensure they were correctly compared to each other. Specifically, each treatment denominator, i.e., total cost or project size, was increased to equal the largest denominator among them. Afterward, the same difference was added to the treatment numerator (39).

Net benefit was determined by summing all benefits and subtracting the sum of all costs of the RAP intervention. This output provides an absolute measure of benefits (total dollars), rather than the relative measures provided by benefit-cost ratio. Incremental net benefit was done. Marginal analysis involved analyzing the relationship between small increases in costs and their impact on output. A critical assumption of this study was that the net benefits, thus benefits and cost, were annualized rather than summed over a specified number of years. This was because the epidemiological study only ran for 18 months. However, the annualized net benefits can provide a basis for future simulations of the present value of RAP with different time periods and discount rates.

Key decision metrics for determining the efficiency of spraying a certain percentage of cattle were the net benefit, benefit-cost ratios, and the incremental benefit-cost ratios. The cut-off benefit-cost ratio was assumed (as is the norm) to equal or exceed the value of 1, as benefits should at least cover, but ideally exceed costs. In a marginal analysis, increasing output (in this case the percentage of cattle sprayed) is considered profitable up to the point at which the marginal cost is either equal to or greater than the marginal benefit. Until then it is considered economical to continue increasing output even though marginal benefits divided by marginal costs may be getting smaller in magnitude (diminishing returns). When the additional benefits received is less than the cost of achieving it, negative returns are experienced, and it is no longer profitable to increase output. Marginal analysis is a widely accepted method of comparing several alternatives with an existing situation (i.e., do nothing, which is T1 in this study) (40).

Costs associated with each treatment included (i) the cost of implementation (delivery cost) incurred By the epidemiological study for the RAP intervention, which has been previously calculated in detail (41), and (ii) the cost incurred by farmers in participating in the RAP intervention, which was derived from this study. The cost of implementation (delivery cost) included full cost of insecticide, trypanocides, and delivering and administering them to the farmers' cattle in their villages. Interviews with farmers in this study enabled their own costs to be collected over the 18-month period, and this was later annualized. The costs to the farmer included time required to collect the animals and to take them for spraying, money spent on ropes, on maintenance of a crush, on hired labor, etc. Combination of the implementation (delivery cost), derived from the epidemiological study, and the farmer related cost of RAP, derived from this study, gave the total societal cost of the intervention.

Benefits associated with each treatment were derived using the gross margin analysis (42). Gross margin analysis allows for household income to be derived. For a livestock enterprise, the gross margin (also known as net revenue) is given by the value of livestock output minus the variable costs of the livestock enterprise (Equation 2). The gross margin is almost always calculated on an annual basis, although this can vary.

(2) $$\begin{array}{r} Gross\;margin\;=\;value\;of\;livestock\;output\;-\;variable\;costs \end{array}$$

In a farm budget, the value of livestock output includes not just production (i.e., the value of both home consumption and sales of milk, animals, and animal traction), but also covers all entries and exits of livestock and the resulting change in herd valuation during the course of the year (26) (Equation 3). Thus, the value of livestock output fully reflects the impact of disease, *via* mortality, fertility, draft days worked, etc.

(3) $$\begin{array}{r} Value\;of\;livestock\;output\;=\;the\;value\;of\;sales,\;transfers\;out\;of\\ \;the\;herd\;and\;home\;consumption\;of\;livestock\;and\\ \;livestock\;products\;-\;the\;value\;of\;livestock\;purchases\\ \;and\;transfer\;into\;the\;herd\;+\;the\;closing\;valuation\;of\\ \;the\;herd\;-\;the\;opening\;valuation\;of\;the\;herd \end{array}$$

Variable costs are defined as those costs that vary with the level of output or are specific to a single farm enterprise (a crop or a livestock product). Importantly, from the animal health viewpoint, all animal health inputs are variable costs, as is feed. The gross margin thus measures the contribution of a specific enterprise to farm income before considering fixed costs, such as housing, rent, farm machinery, and labor, which is not allocated to a specific enterprise. Additionally, gross margin can include value of human labor saved (2) i.e., opportunity cost of labor that could have been incurred if there were no draft cattle and farmers had to plow their own land. The wage rate for computing the human labor saved was obtained from (2).

Annual income per bovine of each livestock keeper was calculated as a gross margin based on the questionnaire data. The change in the livestock keeper's annual income per bovine attributable to treatment was compared to the total annual cost per bovine incurred by the farmers for RAP-related expenses plus the costs incurred by the epidemiological research project for implementing the intervention under each treatment. The latter exclude all purely research costs, but include the full cost of insecticide, trypanocides, and delivering and administering them to the cattle of the farmers in their villages as earlier stated.

Apart from enabling the analysis of the benefit of RAP, gross margin analysis provided a valuable accounting framework for ensuring that all cattle “out” or “in” are accounted for. Monetary data collected from the household questionnaires were in Ugandan shillings, which were converted to United States dollars (USD) at 1 USD being equivalent to 2,575 Ugandan shillings (sourced from OANDA, historical currency exchange rates). It represents the average rate applicable for the period when the study was undertaken and is also the same rate used for calculating the costs of the epidemiological study (40).

## Results

### Intervention Household Characteristics and Cattle Production Parameters

At the beginning of the study, the total number of cattle in the study households was 2,250, with mean and standard deviation (SD) per household of 3.72 and 2.46, respectively. These included 869 draft cattle (38.62%) and 632 cows (28.08%). Additionally, sheep (149), goats (1,222), pigs (808), chickens (5,399), and other animals (184 ducks, 112 turkeys, 27 dogs, and 9 cats) were kept by the farmers. Most livestock are left to forage. The village cattle graze communally within the village. Most households purported to undertake some form of vector control, with only 17.24% reporting that they did nothing. The most common method of vector control for each household was hand tick picking (46.58% of the households), which was also the method most frequently applied, which was on average 11.36 times a year (*n* = 298, min = 2, max = 36, SD = 5.65). Other vector control methods were infrequently used annually, and these included; spraying, 3.57 times (*n* = 194, min = 1, max = 24, SD = 2.88), application of paraffin, 9.61 times (*n* = 23, min = 2, max = 12, SD = 3.69), application of grease, 7.24 times (*n* = 5, min = 4, max = 12, SD = 3.03), and pour-on, 1.13 times (*n* = 14, min = 1, max = 2, SD = 0.36). Of the households that carried out some form of vector control, 22.00% (132) treated work oxen only, 13.83% (83) cows only, and 13.33% (80) heifers only. The cattle types that were treated for ticks and tsetse are summarized in Table 1.

**Table 1:** Types of cattle most often treated for ticks and tsetse flies in each household.

**Type of cattle treated**	**Number of households**	**Number of households in %**
Calf (defined here as young cattle aged 0–2 years) only	0	0.00
Calf and cow (adult female aged 4 years and above)	17	2.83
Calf and heifer (young female aged 2–4 years)	1	0.17
Calf and young male (young male aged 2–4 years)	0	0.00
Calf and non-work oxen	0	0.00
Calf and work oxen	0	0.00
Young male only	15	2.50
Non-work oxen only	7	1.17
Work oxen only	132	22.00
Work oxen and heifer	30	5.00
Work oxen and cow	51	8.50
Heifer only	80	13.33
Heifer and cow	28	4.67
Cow only	83	13.83
Young male and adult male	11	1.83
Young male and heifer	22	3.67
Young male and cow	15	2.50
Young male and calf	5	0.83
None	103	17.17
Total	600	100

The average number of households owning draft cattle was 220 (213 and 227 households at the beginning and the end of the intervention, respectively) out of 600, with 59.09% of these owning two draft cattle and the rest 1, 3, or 4 or more (Table 2). Given that 59.09% of draft cattle owning households had the same number of draft cattle (two oxen), analyzing pooled data from the whole sample was deemed representative. The average days plowed per household over the 18-month study period was 88.13 post-intervention, equivalent to 58.75 days per year (Table 2). Most cattle keepers used their draft cattle to plow farms of other people as a means of generating income. In the year prior to the intervention, the average number of days plowed was 50.46 per year per household among the RAP households.

**Table 2:** Draft oxen ownership and work patterns among the RAP households during the intervention.

		**Average draft oxen days worked per household**		
**Number of work oxen per household**	**% of oxen owning households (*n* = 220)**	**Total plowing days worked**	**Plowing on own farm (SD)**	**Plowing other people's farms (SD)**
1	4.54	96.54	33.76 (12.34)	62.87 (7.61)
2	59.09	87.04	25.53 (10.01)	61.51 (9.04)
3	7.27	82.47	23.94 (9.16)	58.53 (7.37)
4+	29.10	86.48	24.57 (9.81)	61.96 (9.23)
Average recorded over the 18-month study		88.13	26.95	61.21
Adjusted figure for 12 months		58.75	17.96	40.80

### Benefit of RAP to Farmers

Gross margin calculations were undertaken for pooled data for the villages where 25, 50, and 75% cattle were sprayed with deltamethrin using RAP. The items valued included both cash income and expenses and the values of animals not bought or sold and estimated value of farm labor both in components of the variable costs and as an estimate of the value of the labor households saved by using their own work oxen on their own land. The variable costs included: mastitis treatments, the cost of buying and administering trypanocides, spraying against ticks and tsetse, hand-picking ticks, and the cost of borrowing draft cattle from others to plow on the households' own farms. A summary of the cattle numbers and gross margin calculations for T1, T2, T3, and T4 is shown in Table 3.

**Table 3:** Cattle numbers and household gross margin for T1 to T4 during the 18-month period.

**Item**	**Value**							
**Percentage of cattle sprayed using RAP**		**0%**		**25%**		**50%**		**75%**
**Treatment**	**T1 (control)**			**(T2)**		**(T3)**		**(T4)**
Study period*	**Before**	**After**	**Before**	**After**	**Before**	**After**	**Before**	**After**
1. Cattle population (number)								
At start of period	465	456	423	407	481	473	458	451
At end of period	456	440	407	430	473	491	451	485
Average number of cattle during period studied and standard deviation (SD)	461 (2.99)	448 (2.89)	415 (2.84)	419 (3.04)	477 (3.07)	482 (2.93)	455 (3.12)	468 (3.12)
Average per household (95% Confidence Interval)	3.87 (3.24–4.37)	3.66 (3.25–4.14)	3.52 (2.91–3.96)	3.58 (3.01–3.93)	4.00 (3.45–4.56)	4.09 (3.47–4.51)	3.81 (3.20–4.31)	4.04 (3.45–4.34)
2. Gross margin analysis (USD)								
a) Cattle and their produce “out”								
Income from hiring out draft cattle for plowing (SD)	13,694 (139.78)	18,048 (195.00)	15,739 (133.42)	30,503 (243.76)	16,882 (134.77)	33,875 (251.65)	17,046 (130.59)	32,797 (255.69)
Income from hiring out draft cattle for other work (SD)	50 (1.65)	110 (3.03)	54 (1.82)	124 (2.74)	77 (2.01)	176 (2.99)	86 (2.72)	194 (3.71)
Value of cattle sold (SD)	6,290 (124.08)	9,618 (197.62)	9,896 (205.11)	14,130 (219.34)	6,648 (171.48)	16,884 (491.95)	8,016 (181.39)	15,751 (221.33)
Value of cattle given out as loan repayment (SD)	435 (27. 83)	551 (29. 62)	701 (37. 04)	1,181 (44.24)	464 (24. 28)	723 (38. 28)	604 (33. 21)	561 (25. 70)
Value of cattle slaughtered (SD)	384 (35. 06)	518 (33. 33)	593 (38. 54)	933 (46. 04)	389 (26. 12)	476 (30. 68)	453 (29. 34)	679 (35. 63)
Value of human labor saved by using draft cattle (only for cases not involving payment) (SD)	4,797 (47.67)	5,040 (64.96)	5,088 (49.21)	7,386 (72.84)	6,117 (48.47)	7,412 (71.31)	4,749 (44.16)	6,700 (72.92)
Value of milk sold (SD)	1,313 (28.13)	1,614 (47.60)	1,147 (36.98)	2,647 (71.93)	1,381 (43.96)	3,176 (79.19)	1,381 (49.00)	2,917 (99.90)
Subtotal	26,963	35,499	33,218	56,904	31,958	62,722	32,335	59,599
b) Cattle and produce “in”								
Value of draft cattle bought (SD)	3,459 (87.35)	1,176 (51.98)	4,107 (91.73)	6,210 (113.54)	3,927 (83.63)	9,032 (151.72)	4,445 (86.54)	6,980 (116.00)
Value of cattle received as gifts or loan repayment (SD)	1,081 (49.66)	1,224 (73.95)	1,710 (59.89)	1,771 (59.42)	1,349 (56.34)	982 (46. 31)	1,653 (64.47)	906 (48. 99)
Subtotal	4,540	2,400	5,817	7,981	5,276	10,014	6,098	7,886
c) Change in herd value								
Opening valuation (SD)	111,439 (599.99)	109,447 (604.79)	100,610 (600.75)	97,453 (607.70)	116,379 (669.93)	113,120 (656.15)	104,639 (635.17)	102,037 (610.92)
Closing valuation (SD)	109,447 (604.79)	108,479 (607.13)	97,453 (607.70)	97,583 (607.94)	113,120 (656.15)	113,321 (654.77)	102,037 (610.92)	102,285 (610.42)
Change in herd value	−1,992	−968	−3,157	129	−3,259	201	−2,602	248
d) Total livestock output (a–b+c)	20,431	32,131	24,244	49,052	23,423	52,909	23,635	51,961
e) Total variable cost	3,971 (31.66)	3,761 (40.08)	4,947 (35.06)	2,959 (21.62)	6,052 (36.53)	2,004 (19.83)	6,955 (65.69)	1,318 (8. 92)
f) Total gross margin (d–e)	16,460	28,370	19,297	46,093	17,371	50,905	16,680	50,643
g) Total annual gross margin**	16,460	18,914	19,297	30,729	17,371	33,936	16,680	33,762

* *“Before” relates to the 12 months before the intervention began, “after” to the whole of the 18 month intervention period*.

** *The “after” numbers needed to be adjusted from 18- to 12-month estimates*.

Table 3 shows the full data used to calculate the gross margin for the 12 months “before” the intervention and then for the 18 months “after” the intervention began. The observed cattle numbers were similar across the different treatment groups, averaging 455 (range 407–491). The importance of draft power in the cattle economy of the district can be seen by the fact that the single largest item both pre- and post-intervention is income gained from plowing the land of other and labor saved from using draft power for plowing on their own land (Table 3). Overall, the income from hiring out draft cattle plus the value of the human labor saved on their own land comes to 66.9% (range 62.9–72.2%) of the cattle and produce “out” component of livestock output [i.e., item (a) in Table 3]. In addition, for all the treatment groups (i.e., T1 to T4) in the absence of RAP, the value of the herd was lower at the end than at the beginning of the period due to reduced cattle population (mostly from cattle mortality) before the intervention.

For the further analyses, the “after” figures collected during the intervention were converted from 18 months to 12, by dividing them by 1.5 to be comparable with the “before figures.” The difference in the annual variable cost per all bovine before and after the intervention was USD 3.16, USD 7.24, USD 9.91, and USD 13.44 for T1, T2, T3, and T4, respectively. This reduction in variable costs was mostly due to reductions in expenditure on trypanosomiasis and vector control. The slight drop in the variable cost for the control was probably from the wider impact of RAP on tsetse and tick populations.

To obtain the mean annual income per bovine in the RAP and control households, the changes in gross margins were obtained from Table 3 and divided by the number of households (i.e., 120 households in each treatment). The mean annual income from livestock per RAP household ranged from USD 95.26–USD 142.35, a mean of USD 125.22 across all the RAP households, whereas the mean increases in annual income per bovine in the RAP treatment varied between USD 26.93 and USD 35.44 (mean of USD 32.12 across all the RAP households) using the average cattle population for each treatment as the denominator (Table 4). However, an increase in the annual income of USD 6.51 was also observed in the T1 households (which only received an initial trypanocide treatment).

**Table 4:** Annual cattle gross margin and benefit from using RAP compared to control.

**Percentage sprayed using RAP (treatment)**	**Total annual cattle gross margin across households (USD)**		**Difference in annual cattle gross margin across all households (USD)**		**Cattle population per treatment (SD)**		**Difference in annual cattle gross margin per bovine (USD)**
	**Before intervention**	**After intervention**	**Difference all households**	**Mean per household (*n* = 120 per treatment)**	**Before intervention**	**After intervention**	Mean per* bovine
0% (T1)	16,460	18,914	2,454	20.45	461 (2.99)	448 (2.89)	6.51
25% (T2)	19,297	30,729	11,432	95.26	415 (2.84)	419 (3.04)	26.93
50% (T3)	17,371	33,936	16,565	138.04	477 (3.07)	482 (2.93)	33.99
75% (T4)	16,680	33,762	17,082	142.35	455 (3.12)	468 (3.12)	35.44

* *Obtained by first, dividing the gross margins and the cattle population for each treatment, before and after the intervention, and then subtracting these*.

### Cost of RAP and Analysis of Benefits and Costs

The mean number of cattle that were sprayed using RAP across the 360 RAP intervention households was 1,406, with each household having an average of 3.84 (SD 3.76) cattle within the 18-month period. The average number of times farmers took their cattle for monthly spraying was 16.24 (of a possible 18 times) representing a compliance of 90%. The total cost incurred by all farmers participating in the treatments during the 18 months is summarized in Table 5. This shows a nearly four-fold increase in these cost items between T1 (no RAP but cattle were periodically gathered for biophysical monitoring) and T2 (25% of animals sprayed), increasing for T3 and T4 due to higher proportions of cattle sprayed. The main increases were the cost of labor and expenditure on ropes.

**Table 5:** Livestock keepers' expenditure on intervention related items over 18 months.

	**Treatments (USD)**			
**Item**	**T1**	**T2**	**T3**	**T4**
Value of farmers' time taking cattle for RAP and/or trypanocide treatment	27	121	151	205
Payment to casual laborers/herdsmen	3	2	4	5
Cash spent on ropes	8	29	50	60
Crush repair	4	4	5	8
Payment to someone help restrain cattle	0	0	1	2
Payment for water to mix pyrethroids	0	2	4	5
Total expenditure	42	159	216	286
Cattle population	448	419	482	468
Expenditure per bovine per year	0.06	0.25	0.30	0.41

The total societal cost of RAP per year was estimated to be USD 4.33, USD 6.11, and USD 7.94 per bovine per year for spraying 25, 50, and 75% of cattle, respectively. This figure was derived from (1) costs of the RAP component only (i.e., spraying of cattle with insecticide only and excluding administration of Veriben B12), which came to USD 2.02, 3.75, and 5.47 per bovine per year for spraying 25, 50, and 75% of cattle, respectively (41), (2) administering Veriben B12 (41), and (3) cost incurred by farmers when taking their cattle for spraying and administration of Veriben B12, which was obtained from this study.

**Figure 1:**
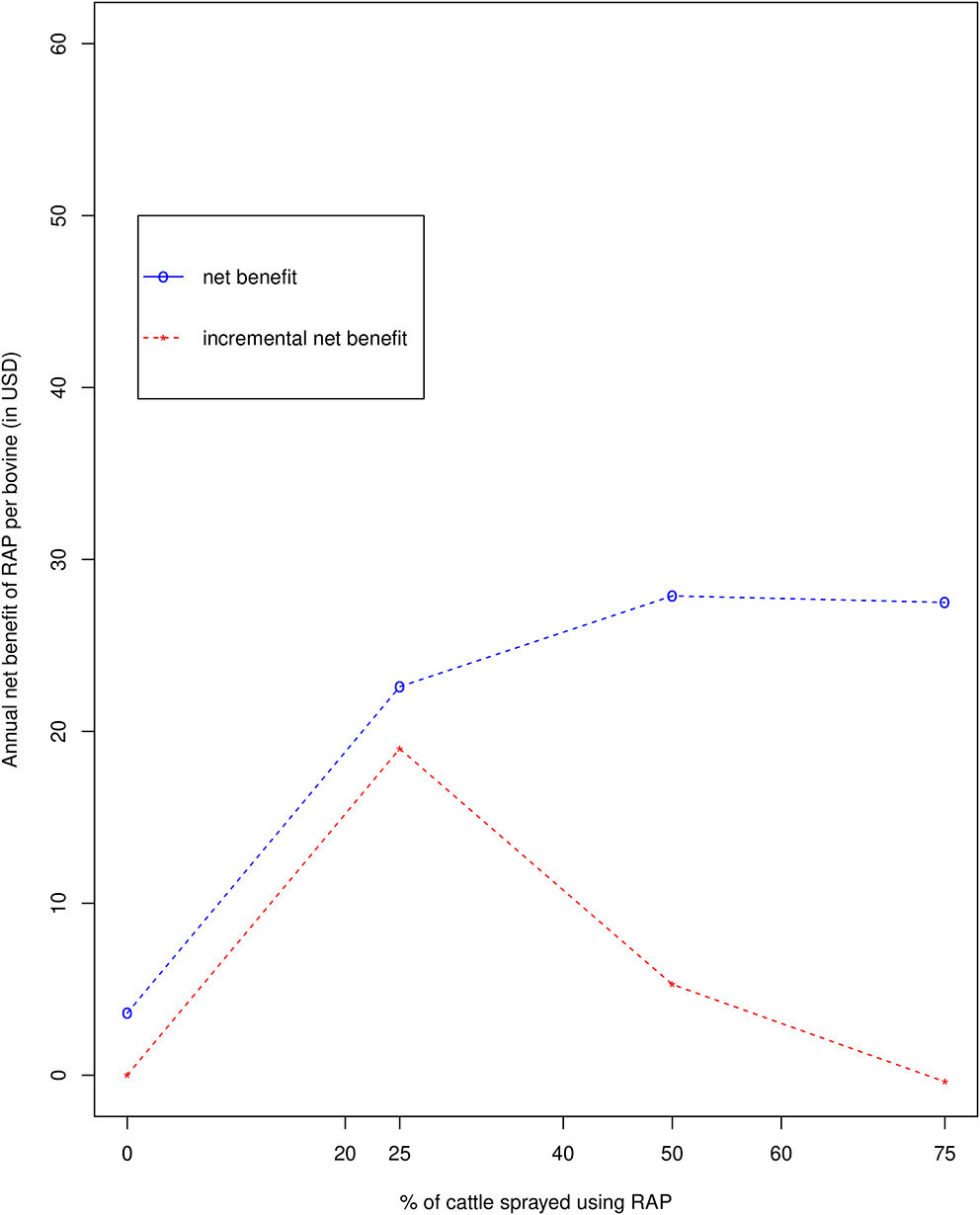
Average and incremental net benefits for each treatment regime.

In the cost analysis of the RAP intervention undertaken as part of the epidemiological study (41), the full costs incurred were calculated, including all staff costs, overheads and depreciation. The average cost per bovine of Veriben B12, needles, syringes and sterile water was USD 0.81 per dose (41). For the RAP treatments, where the Veriben B12 drug was administered at the same time as the RAP treatment, the need to include veterinarians for administering the drug was estimated to add USD 0.22 to the delivery cost of USD 0.39 per bovine for RAP alone, also based on the figures in the RAP cost analysis (41). For T1, the total societal cost incurred was only from administration of Veriben B12, and this was estimated to be USD 0.81 per dose (41), with additional delivery cost of USD 0.61 per dose (41). Administering Veriben B12 thus incurred a cost of USD 2.84 in T1 and USD 2.06 per bovine in T2, T3, and T4. The additional expenditure of the farmers were added to the Veriben B12 costs and the RAP epidemiological research project costs cited above to obtain the total annual societal cost of the intervention. For T1, the total cost of Veriben B12 and the slight increase in farmers' costs came to USD 2.90 per bovine, which when set against the increase in income of USD 6.51 yielded a net benefit of USD 3.61, and a benefit-cost ratio of 1.55 when adjusted using the highest intervention cost which was USD 7.94 for T4 (39). The marginal benefits and costs are calculated as the changes in benefits and costs attributed to increasing the proportion of the cattle herd sprayed, i.e., moving from one treatment to the next (Table 6). The annual net benefit for treatments T1, T2, T3, and T4 was USD 3.61, USD 22.60, USD 27.88, and USD 27.50, respectively as shown in Table 6. The benefit-cost analysis of spraying 25%, 50% and 75% of the cattle population yielded average benefit-cost ratios of 3.85, 4.51, and 4.46 for T2, T3, and T4, respectively as shown in Table 6. The incremental benefit-cost ratios from spraying each additional 25% of the population cattle were 11.38, 3.89, and 0.79, respectively (Table 6), showing a very high return on investment for spraying 50% of the population, with returns reducing thereafter. Also, the farmers benefit-cost ratio when 50% of the cattle are sprayed was 113.30, obtained by dividing the total benefits at 50% by the cost incurred by farmers at the same level (Table 6). Figure 1 illustrates the annual net benefit and the incremental benefit in USD from spraying each additional 25% of the population with returns reducing thereafter.

**Table 6:** Benefits and costs of different treatments per year per bovine owned.

**Proportion of cattle sprayed using RAP (in %)**	**Benefit (in USD)**		**Cost (in USD)**					**Benefit-cost and incremental analysis**				
	**Total benefit**	**Marginal benefit**	**Cost of spraying cattle using RAP**	**Cost of Veriben B12 (double injection)**	**Cost incurred by the farmers**	**Total intervention cost**	**Marginal cost**	**Net benefit**	**Incremental net benefit**	**Uncorrected benefit-cost ratio**	**Corrected benefit-cost ratio**	**Incremental Benefit-cost ratio**
0% (T1)	6.51	–	0	2.84	0.06	2.90	–	3.61	–	2.24	1.55	–
25% (T2)	26.93	20.42	2.02	2.06	0.25	4.33	2.21	22.6	18.99	6.22	3.85	11.38
50% (T3)	33.99	7.06	3.75	2.06	0.3	6.11	1.78	27.88	5.28	5.56	4.51	3.89
75% (T4)	35.44	1.45	5.47	2.06	0.41	7.94	1.83	27.5	−0.38	4.46	4.46	0.79

## Discussion

This is the first comprehensive analysis of the impact of AAT to use a large sample size and longitudinal survey to focus on farm incomes directly rather than on underlying cattle production parameters (such as mortality, fertility, weight, etc.). The most similar investigation was the multi-site African Trypanotolerant Livestock Network which linked production parameters to trypanosomiasis status on individual animal basis, as well as to study sites and herds (23).

The intervention studied here was innovative in including both a control group, different levels of Control, and a before and after comparison of the 480 households across 16 villages, excluding T5, followed longitudinally. This meant that, unlike studies that look only at either with/without intervention or before/after intervention, by looking at both, the present analysis, as far as possible, removes the likelihood of outside factors influencing the outcomes. Before/after studies run the risk of time-related changes, such as better rainfall after the intervention, which may bias the results. With/without studies run the risk that all participating households become more focused on improving the management of their animals. This latter risk was mitigated in this study by comparing different regimes. The no RAP control did show some benefits, possibly linked to taking part in the study or, more likely, to the production benefits from the initial trypanosomiasis treatment since trypanosomiasis infection levels did not regain their pre-intervention levels during the study period (28). The marginal analyses removed even that source of potential bias, by analyzing the incremental benefits and costs ratios for each proportion of cattle herd sprayed as part of the RAP intervention. The gross margin format worked well, both as a checklist for data collection and as a basis for analyzing the farm-level impacts of the intervention. It is a classic tool for analyzing farm level profitability of individual enterprises, in this case, cattle keeping. However, it does not include the fixed costs of the farms (such as rent, labor allocated to all farm enterprises, and machinery costs). In this analysis, all the relevant labor costs for the cattle enterprise were included in the gross margin, having been discussed in the questionnaire, which focused on time inputs and valued these at local labor rates. The only enterprise-specific fixed cost was depreciation on plows, which was unaffected by the intervention. While cattle numbers and output from cattle increased in all the RAP treatments, variable costs decreased, increasing the farm gross margin associated with each treatment. There are several possible spillover benefits from the intervention that we did not necessarily measure in this study. The use of ITC may result in farmers focusing on better management of their cattle as well as reducing the occurrence of AAT in other livestock species: equines, sheep, goats, and pigs. In the study area, cattle, sheep, and goats are the only livestock whose productivity is significantly affected by *T. vivax* and *Trypanosoma congolense*, with *T. brucei* having little effect on livestock health. Usually valued at 0.1 tropical livestock units each, the biomass of the small ruminant population recorded for the study households would be about 6% of that of their cattle, which would be reflected in the income they generated. It is likely that there were some benefits to small ruminant health *via* reduced exposure to tsetse fly bites, although, as discussed below, tsetse feed preferentially on larger animals. Similarly, reducing tsetse and tick populations will benefit non-participating livestock of households. Spillover benefits for human health from RAP, by reducing transmission of zoonotic rHAT were outside the scope of this study. However, the One Health implications of treating cattle with trypanocides and RAP and impact on rHAT have been described and modeled previously (27, 32, 43). Mass chemoprophylaxis and RAP applied to 500,000 cattle between 2006 and 2008 was accompanied by a reduction of 75% in the animal trypanosome prevalence, accompanied by a 90% reduction in reported rHAT cases (43). Additionally, administration of a single dose of diminazene aceturate by the Stamp out Sleeping Sickness public-private partnership in 2008 in districts to the north of Tororo saw a reduction in the prevalence of *T.b. rhodesiense* in cattle from 2.4 to 0.74% (32).

Investing in spraying 50% of cattle provides the highest average return with a net benefit of USD 27.88 per bovine per year and indicates that changing from a scenario where farmers practiced minimal vector control to spraying 50% of cattle using RAP offered the best returns with an incremental net benefit still greater than zero (i.e., marginal benefit approaching marginal cost). When a further 25% (i.e., 75%) of the cattle population was sprayed, marginal costs exceeded marginal benefits, a negative incremental net benefit of USD −0.38 per bovine per year with negative returns was observed. This study suggests a higher proportion of the cattle to be sprayed compared to the prediction of the earlier modeling study that spraying 27% of the cattle population would be sufficient to control *T. brucei s.l*. (27). This level of spraying would be expected to be sufficient to lower the risk of transmission of zoonotic rHAT from animals to people while, as demonstrated here, providing an attractive benefit to cattle keepers. These findings do, however, contrast with the observations for trypanosomiasis prevalence (28) where an inverse relationship between dose (increase in RAP coverage; 25% RAP, 50% RAP, and 75% RAP) and reduction in trypanosome prevalence was observed (28). The authors of (28) focused on prevalence as opposed to the wider production benefits evaluated in our study.

The work described here shows that increasing the proportion of the herd sprayed does increase income per bovine, but with diminishing returns. Investments in additional inputs should continue if these incremental investments yield a positive net benefit, or an incremental benefit-cost ratio greater than one. Theoretically, investment should continue up to 50% (the highest net benefit).

This aligns with the preferences of farmers for treating work oxen and cows, which, respectively, account for 38.62 and 28.08% of the cattle population. Tsetse feed preferentially on larger animals, so the effect of spraying a selected 50% subset of their herds may be expected to have an enhanced effect on tsetse populations (44) and even benefit non-participants since tsetse control, in the community, is seen as a public good (9).

Additionally, characteristics of the intervention households indicate that there are probably some households that have specialized in keeping draft oxen providing services to farms of other people. Other studies in south-east Uganda have also found this to be the case (21, 22). The average increase in income per bovine over the three RAP regimes deployed in this study was USD 32.12, a figure in keeping with other estimates. The modeled increase in average annual income per bovine in agro-pastoral and mixed farming high-oxen use systems in East Africa in the absence of trypanosomiasis was previously calculated at USD 20 and 25, respectively when converted to year 2015 USD values (25).

For the RAP intervention in Uganda, where the research project paid for the bulk of costs, looking only at the farmers' costs, the implied average benefit-cost ratio to the farmers would be 113.30 for 50% RAP. This high figure would change if livestock keepers bore some of the delivery and insecticide costs (e.g., sourcing and applying the insecticide which accounted for just over 20% of total costs of RAP). There has been an encouraging uptake of RAP by cattle keepers in areas to the north and west of Tororo District, with nearly 800,000 doses of RAP being applied to cattle in 2016/2017 (45) and whereby insecticide was sold to local operators, who delivered RAP to farmers with some subsidies.

The opportunity cost of cattle keepers' labor (a non-cash item) and buying ropes (a cash item) were two of the most significant expenditures incurred by the farmers during the RAP intervention, accounting for 92.65% of their expenses. Farmers forgo certain activities such as herding, planting, harvesting, socializing, etc. to participate in spraying. Moreover, they have to gather the cattle, take them for spraying, participate in the spraying, and then bring them back to the homestead. There are few communal crushes in Tororo District and so farmers frequently required ropes for tethering and restraint during spraying and substantial amounts of cash buying ropes. The location selected for application of spraying and efficiency in restraining cattle are critical factors to consider in communal spraying since they have a major influence on the cost incurred by the farmer. Such information could be used to lobby for communal crushes.

## Conclusions

Based on household interviews at 6-month intervals, this study compared the costs and benefits of spraying different proportions of the cattle population using RAP both to the pre-intervention situation and to a control, thus looking at before and after as well as with or without intervention. Spraying 50% of the cattle yielded the highest net benefit. Increasing the proportion of cattle sprayed using RAP led to an increased income per bovine but with diminishing returns, and spraying of 75% of cattle no longer yielded a positive incremental benefit, as marginal costs outweighed marginal benefits.

Spraying only the most valuable adult cattle using insecticides effective against tsetse may be the most cost-effective measure for farmers; in this part of Uganda, these would be draft cattle comprising 38.62% of the herd approaching the threshold of 50% that yielded the highest incremental benefit-cost ratio. In addition, most farmers in this area would preferentially treat their draft cattle.

## Data Availability Statement

The original contributions presented in the study are included in the article/supplementary material, further inquiries can be directed to the corresponding author/s.

## Ethics Statement

The studies involving human participants were reviewed and approved by Uganda National Council for Science and Technology and approved under approval number HS1336. The patients/participants provided their written informed consent to participate in this study.

## Author Contributions

WO was responsible for conception, design, collection, drafting, and analysis of data. DM was involved in design and data collection. EM was involved in design and drafting of the manuscript. SW and CW were involved in the inception of the study, study design, and revising the intellectual content. All authors read and approved the final version of the manuscript.

## Conflict of Interest

The authors declare that the research was conducted in the absence of any commercial or financial relationships that could be construed as a potential conflict of interest.

## Abbreviations

AAT: animal African trypanosomiasis
rHAT: human African trypanosomiasis caused by *T. b. rhodesiense*
ITC: insecticide-treated cattle
RAP: restricted application protocol
TBDs: tick-borne diseases
SD: standard deviation
USD: United States Dollar.
